# Supplementary material for: Overcoming the impacts of two-step batch effect correction on gene expression estimation and inference
Source: Biostatistics. 2021 Dec 10;24(3):635–52. doi: 10.1093/biostatistics/kxab039 (PMC10449015; doi:10.1093/biostatistics/kxab039)
Supplement: kxab039_Supplementary_Data [file kxab039_supplementary_data.pdf]

Biostatistics (2021), **0**, 0, *pp.* 1–7
doi:10.1093/biostatistics/output

Overcoming the impacts of two-step batch effect correction on gene expression estimation and inference
Supplementary Material

TENGLONG LI, YUQING ZHANG, PRASAD PATIL, W. EVAN JOHNSON*,

Academy of Pharmacy, Xi'an Jiaotong-Liverpool University, Suzhou, Jiangsu, China, Division of Computational Biomedicine, School of Medicine, Boston University, Boston, MA, USA and Department of Biostatistics, School of Public Health, Boston University, Boston, MA, USA

wej@bu.edu

1. Derivation of the sample covariance matrix for the two-step approach

Based on the Frisch-Waugh-Lovell theorem (Frisch and Waugh, 1933; Lovell, 1963, 2008), the estimate for batch effect $\hat{\beta}_{2g}$ in the regression model $Y_g = X_1\beta_{1g} + X_2\beta_{2g} + \epsilon_g, \epsilon_g \sim N(0, \sigma_g^2 I)$, is the same as the $\hat{\beta}_{2g}$ in the following regression model:

$$PY_g = PX_2\beta_{2g} + \epsilon_g, \epsilon_g \sim N(0, \sigma_g^2 I) \tag{1.1}$$

where $P = I - X_1(X_1^T X_1)^{-1} X_1^T$. We will also have $\hat{\beta}_{2g} = (X_2^T P X_2)^{-1} X_2^T P Y_g$.

Model (1.1) the first step of the two-step batch adjustment. In the second step of the two-step batch adjustment, the batch adjusted data $\tilde{Y}_g$ is obtained as:

$$\tilde{Y}_g = Y_g - X_2\hat{\beta}_{2g} = (I - X_2(X_2^T P X_2)^{-1} X_2^T P)Y_g = (I - H_{12})Y_g \tag{1.2}$$

*To whom correspondence should be addressed.

© The Author 2021. Published by Oxford University Press. All rights reserved. For permissions, please e-mail: journals.permissions@oup.com

where H_{12} is:

$$H_{12} = X_2(X_2^T P X_2)^{-1} X_2^T P = X_2(X_2^T(I - X_1(X_1^T X_1)^{-1} X_1^T) X_2)^{-1} X_2^T (I - X_1(X_1^T X_1)^{-1} X_1^T) \tag{1.3}$$

Therefore, the batch adjusted data has covariance matrix $\sigma_g^2(I - H_{12})(I - H_{12})^T$.

It is noteworthy that X_1 should also include the all-ones vector $\mathbf{1}$ when there is an reference batch. That is $H_{12} = X_2(X_2^T(I - X_0(X_0^T X_0)^{-1} X_0^T) X_2)^{-1} X_2^T (I - X_0(X_0^T X_0)^{-1} X_0^T)$ where $X_0 = [\mathbf{1}, X_1]$.

2. The relationship between biological effect estimates and batch design

In this section, we will show the relationship between biological effect estimates and batch design. Without loss of generality, the regression model (for each gene) is formulated as $Y = \alpha + X_1\beta_1 + X_2\beta_2 + \epsilon, \epsilon \sim N(0, \sigma^2 I)$, where α is the background gene expression. X_1 represents the biological groups (assume there are two biological groups) and X_2 represents the batch design. Furthermore, we define the matrix $X = [X_2, X_1]$ and the matrix $V = [\mathbf{1}, X]$. We also define sample variance $\hat{\sigma}_{xx}$ and covariances $\hat{\sigma}_{xy}$ as follows:

$$\hat{\sigma}_{xx} = \frac{1}{n}\sum_{i=1}^{n}(x_i - \bar{x})^2 \tag{2.4}$$

$$\hat{\sigma}_{xy} = \frac{1}{n}\sum_{i=1}^{n}(x_i - \bar{x})(y_i - \bar{y}) \tag{2.5}$$

Our goal is to derive the least square estimate $\hat{\beta}_1$ and its variance based on the sample variance-covariance matrix of X. It's known that the least square estimate has the matrix form $(V^T V)^{-1} V^T Y$. Specifically, $V^T V$ is the following block matrix:

$$V^T V = \begin{pmatrix} n & n\bar{X}^T \\ n\bar{X} & X^T X \end{pmatrix} \tag{2.6}$$

where $\bar{X} = [\bar{X_2}, \bar{X_1}]^T$.

The inverse of V^TV then becomes:

$$(V^TV)^{-1} = \begin{pmatrix} n^{-1} + n^{-1}\bar{X}^T S_{XX}^{-1}\bar{X} & -n^{-1}\bar{X}^T S_{XX}^{-1} \\ -n^{-1}S_{XX}^{-1}\bar{X} & n^{-1}S_{XX}^{-1} \end{pmatrix} \tag{2.7}$$

Furthermore, the covariance matrix S_{XX} is the following block matrix:

$$S_{XX} = \begin{pmatrix} S_{22} & S_{21} \\ S_{12} & \hat{\sigma}_{11} \end{pmatrix} \tag{2.8}$$

where S_{22} is the covariance matrix of X_2, S_{21} is the covariance matrix between X_2 and X_1 (b rows and 1 column; b is the number of batch indicators), and S_{12} is just the transpose of S_{21}. The inverse of S_{XX} is:

$$S_{XX}^{-1} = \begin{pmatrix} S_{22}^{-1} + S_{22}^{-1}S_{21}(\hat{\sigma}_{11} - S_{12}S_{22}^{-1}S_{21})^{-1}S_{12}S_{22}^{-1} & -S_{22}^{-1}S_{21}(\hat{\sigma}_{11} - S_{12}S_{22}^{-1}S_{21})^{-1} \\ -(\hat{\sigma}_{11} - S_{12}S_{22}^{-1}S_{21})^{-1}S_{12}S_{22}^{-1} & (\hat{\sigma}_{11} - S_{12}S_{22}^{-1}S_{21})^{-1} \end{pmatrix} \tag{2.9}$$

Plugging in the above expression of S_{XX}^{-1} into the block matrix in (2.7) will give the complete form of $(V^TV)^{-1}$, whose elements are all sample means or sample variances/covariances. To isolate $\hat{\beta}_1$, only the last row of $(V^TV)^{-1}$ is needed:

$$(V^TV)^{-1}_{(b+2)1} = n^{-1}[(\hat{\sigma}_{11} - S_{12}S_{22}^{-1}S_{21})^{-1}S_{12}S_{22}^{-1}\bar{X}_2 - \bar{X}_1(\hat{\sigma}_{11} - S_{12}S_{22}^{-1}S_{21})^{-1}] \tag{2.10}$$

$$[(V^TV)^{-1}_{(b+2)2}, \ldots, (V^TV)^{-1}_{(b+2)(b+1)}] = -n^{-1}(\hat{\sigma}_{11} - S_{12}S_{22}^{-1}S_{21})^{-1}S_{12}S_{22}^{-1} \tag{2.11}$$

$$(V^TV)^{-1}_{(b+2)(b+2)} = n^{-1}(\hat{\sigma}_{11} - S_{12}S_{22}^{-1}S_{21})^{-1} \tag{2.12}$$

$\hat{\beta}_1$ is straightforward given the following expression of V^TY:

$$V^TY = \begin{pmatrix} n\bar{Y} \\ nS_{2Y} + n\bar{Y}\bar{X}_2 \\ n\hat{\sigma}_{1Y} + n\bar{X}_1\bar{Y} \end{pmatrix} \tag{2.13}$$

The expression of $\hat{\beta}_1$ is then:

$$\hat{\beta}_1 = \frac{\hat{\sigma}_{1Y} - S_{12}S_{22}^{-1}S_{2Y}}{\hat{\sigma}_{11} - S_{12}S_{22}^{-1}S_{21}} \tag{2.14}$$

and its variance is:

$$\text{Var}(\hat{\beta}_1) = \frac{\hat{\sigma}^2}{n}(\hat{\sigma}_{11} - S_{12}S_{22}^{-1}S_{21})^{-1} \tag{2.15}$$

where $\hat{\sigma}^2$ is the estimated residual variance in regression.

3. The relationship between H_{12} and S_{12}

Without loss of generality, we assume there is a reference batch and thus $H_{12} = X_2(X_2^T(I - X_0(X_0^TX_0)^{-1}X_0^T)X_2)^{-1}X_2^T(I - X_0(X_0^TX_0)^{-1}X_0^T)$ where $X_0 = [\mathbf{1}, X_1]$. Following the regression framework in the previous section, we can derive the expression of $(X_0^TX_0)^{-1}X_0^TX_2$. First, we have:

$$(X_0^TX_0)^{-1} = \begin{pmatrix} n^{-1} + n^{-1}\bar{X}_1^2\hat{\sigma}_{11}^{-1} & -n^{-1}\bar{X}_1\hat{\sigma}_{11}^{-1} \\ -n^{-1}\bar{X}_1\hat{\sigma}_{11}^{-1} & n^{-1}\hat{\sigma}_{11}^{-1} \end{pmatrix} \tag{3.16}$$

and:

$$X_0^TX_2 = \begin{pmatrix} n\bar{X}_2 \\ nS_{12} + n\bar{X}_1\bar{X}_2 \end{pmatrix} \tag{3.17}$$

where $\bar{X}_2 = [\bar{X}_{21}, \bar{X}_{22}, \ldots, \bar{X}_{2b}]$, a 1 by b vector whose elements are means of the batch indicators in X_2.

Taken together, we have the expression of $(X_0^TX_0)^{-1}X_0^TX_2$ as follows:

$$(X_0^TX_0)^{-1}X_0^TX_2 = \begin{pmatrix} \bar{X}_2 - \bar{X}_1\hat{\sigma}_{11}^{-1}S_{12} \\ \hat{\sigma}_{11}^{-1}S_{12} \end{pmatrix}_{2\times b} \tag{3.18}$$

3.1 *Special case: balanced designs*

For a balanced group-batch design, the elements in S_{12} are all 0, which means:

$$(X_0^TX_0)^{-1}X_0^TX_2 = \begin{pmatrix} \bar{X}_2 \\ \mathbf{0} \end{pmatrix}_{2\times b} \tag{3.19}$$

Based on (3.19), we can derive the expression of $(I - X_0(X_0^TX_0)^{-1}X_0^T)X_2$ as follows:

$$(I - X_0(X_0^TX_0)^{-1}X_0^T)X_2 = \begin{pmatrix} X_{211} - \bar{X}_{21} & X_{212} - \bar{X}_{22} & \cdots & X_{21b} - \bar{X}_{2b} \\ X_{221} - \bar{X}_{21} & X_{222} - \bar{X}_{22} & \cdots & X_{22b} - \bar{X}_{2b} \\ \vdots & \vdots & \cdots & \vdots \\ X_{2n1} - \bar{X}_{21} & X_{2n2} - \bar{X}_{22} & \cdots & X_{2nb} - \bar{X}_{2b} \end{pmatrix}_{n\times b} \tag{3.20}$$

It is clear that (3.20) is just the centered version of X_2 (denoted as X_2^c), and so we can express H_{12} as:

$$H_{12} = X_2(X_2^TX_2^c)^{-1}(X_2^c)^T \tag{3.21}$$

With H_{12} in the form of (3.21), the correlation between two samples from two different batches in the matrix M is $\frac{1}{n_r}$ where n_r is the sample size of the reference batch. If a reference batch is not used, as is the case with most applications of ComBat, the correlations are not as straightforward, but can be derived using a similar procedure, and lead to the same conclusion (covariance only depends on the batch design).

To summarize, when the group-batch design is balanced, H_{12} is solely a function of the batch design X_2 and has nothing to do with the group design X_1. Therefore, removing batch effects in the first step won't result in the *endogeneity* issue for the second step. When the group-batch design is unbalanced, H_{12} depends on both the batch design X_2 and the group design X_1. The relationship between H_{12} and S_{12} (and thus X_1) can be derived by plugging the expression (3.18) into the expression of H_{12}, which will not be detailed here. Most importantly, when the group-batch design is unbalanced, the covariance vector S_{12} will not be $\mathbf{0}$ and thus H_{12} will depend on X_1 and the strength of such dependence is characterized by S_{12}.

4. Additional analysis of the example 4

We ran a simulation based on the example 4: progressors versus non-progressors in tuberculosis and compared the performances of ComBat, ComBat+Cor, SVA and RUV. The results are presented in Table 1 and Figure 1.

References

Frisch, Ragnar and Waugh, Frederick V. (1933). Partial time regressions as compared with individual trends. *Econometrica: Journal of the Econometric Society* **1**(4), 387–401.

Lovell, Michael C. (1963). Seasonal adjustment of economic time series and multiple regression analysis. *Journal of the American Statistical Association* **58**(304), 993–1010.

Lovell, Michael C. (2008). A simple proof of the fwl theorem. *The Journal of Economic Education* **39**(1), 88–91.

[]

Table 1. *The false positive rates (FPR) and the true positive rates (TPR) of the methods in comparison for the simulation based on the example 4: progressors versus non-progressors in tuberculosis.*

Approach	FPR	TPR
T-test	17.5%	96.6%
Benchmark	5.0%	96.8%
One-step	4.4%	96.5%
ComBat	1.9%	96.8%
ComBat+Cor ($\zeta = 0.1\%$)	1.1%	96.4%
SVA	10.9%	86.5%
RUV	16.5%	96.5%

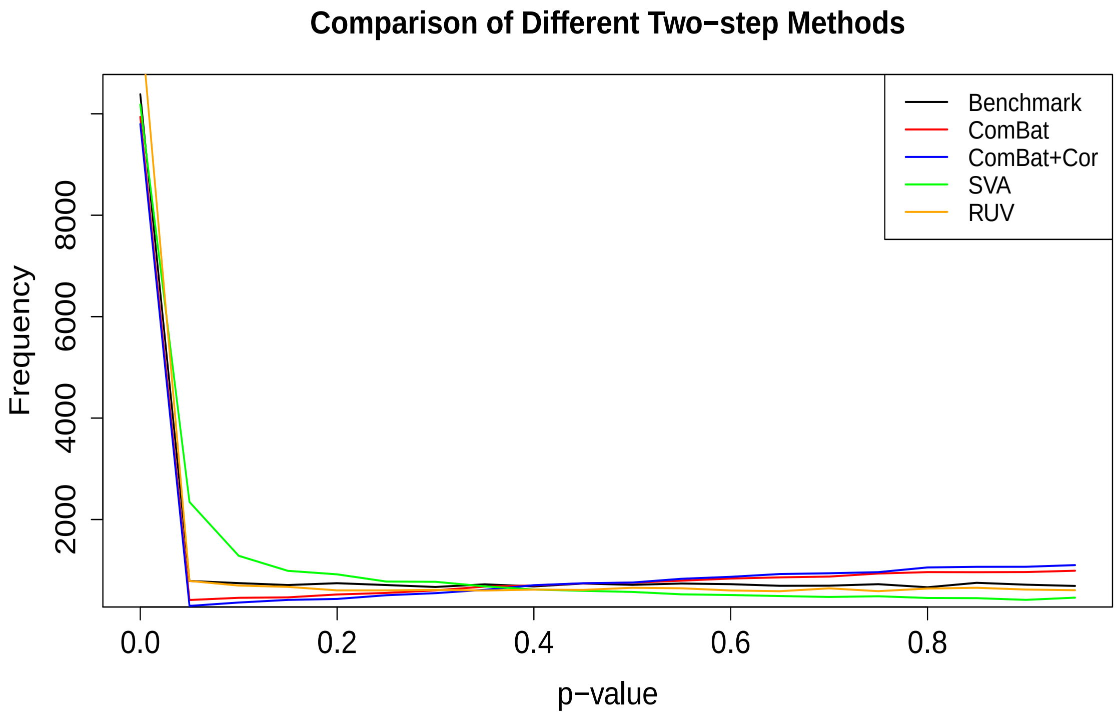

Fig. 1. Distributions of the p-values for the methods in comparison for the simulation based on the example 4: progressors versus non-progressors in tuberculosis.
